# Study of the Properties of In Vitro *Dactylorhiza maculata* (L.) Soó (Family *Orchidaceae*) Extracts

**DOI:** 10.3390/plants10071330

**Published:** 2021-06-29

**Authors:** Stanislav Sukhikh, Svetlana Noskova, Svetlana Ivanova, Liubov Skrypnik, Artem Pungin, Elena Ulrikh, Evgeny Chupakhin, Olga Babich

**Affiliations:** 1 Institute of Living Systems, Immanuel Kant Baltic Federal University, A. Nevskogo Street 14, 236016 Kaliningrad, Russia; stas-asp@mail.ru (S.S.); svykrum@mail.ru (S.N.); LSkrypnik@kantiana.ru (L.S.); apungin@kantiana.ru (A.P.); chupakhinevgen@gmail.com (E.C.); olich.43@mail.ru (O.B.); 2 Department of Bionanotechnology, Kemerovo State University, Krasnaya Street 6, 650043 Kemerovo, Russia; 3 Natural Nutraceutical Biotesting Laboratory, Kemerovo State University, Krasnaya Street 6, 650043 Kemerovo, Russia; 4 Department of General Mathematics and Informatics, Kemerovo State University, Krasnaya Street 6, 650043 Kemerovo, Russia; 5 Kuzbass State Agricultural Academy, Markovtseva Street 5, 650056 Kemerovo, Russia; elen.ulrich@mail.ru

**Keywords:** *Dactylorhiza maculata*, extracts, biologically active substances, toxicological, antimicrobial properties, antioxidant activity

## Abstract

The medicinal plant *Dactylorhiza maculata* (L.) Soó (family *Orchidaceae*) is used to treat gastritis, colic, gastrointestinal tract, and bladder diseases. This study aimed to investigate the properties and characteristics of the in vitro *Dactylorhiza maculata* extract. The recommended parameters for producing *Dactylorhiza maculata* extract were determined: temperature 60 °C, process duration 60 min, hydro module 1:10. It is recommended to carry out the extraction using an aqueous ethyl alcohol solution with a mass fraction of the parent substance of 70%. It was found that such biologically active substances as rutin, quercetin, 3,3′,4′,5,5′,7-hexahydroxyflavonone, 3,3′,4′,5,5′,7-hexahydroxyflavonone-3-*O*-glycoside, gallic acid, and ferulic acid were dominant in *Dactylorhiza maculata.* A high phosphorus content was noted (2410.8 mg/kg dry matter). The studied *Dactylorhiza* *maculata* samples contained a large number of organic acids and water-soluble vitamins. The tested extracts were safe in terms of the content of heavy metals, pesticides, aflatoxin B1, and radionuclides, as well as pathogenic and opportunistic microorganisms; the content was significantly lower than the threshold limit values. The studied complex of biologically active substances from *Dactylorhiza maculata* extract samples had antimicrobial properties. It was found that the antioxidant activity of the samples was 217.89 ± 10.89 mg AA/g (AA—ascorbic acid). The high content of bioactive substances and the antimicrobial and antioxidant properties of *Dactylorhiza* *maculata* extract samples determine the application potential of this plant as a substitute for growth stimulants and feed antibiotics in the production of feed additives, aiming to increase the physiological and immune status of livestock and poultry.

## 1. Introduction

Gastrointestinal diseases from various causes present a considerable problem for the livestock industry, as they lead to low productivity and the death of young animals [1]. Some of the factors or reasons for dysfunctions of the gastrointestinal system are closely connected to aspects of industrial livestock production, and include the stresses associated with veterinary treatments, housing, and feeding conditions, including the quality of feed [2].

Among the many medicines used to preserve the livestock and treat diseases of the gastrointestinal tract, course and feed antibiotics are most often used; however, their prolonged and unconsidered use reduces the body’s resistance to fungal and staphylococcal infections, and disrupts the processes of digestion and metabolism, as a result of which it decreases live weight gain and the quality of livestock products, including the fact that meat raw materials may contain residual amounts of antibiotic agents [3]. In addition, pathogens tend to acquire resistance to frequently and long-used antibiotics, leading to the ineffectiveness of their use [4].

Currently, the number of studies on the use of medicinal plants in the treatment and prevention of a wide range of diseases in humans and animals has increased worldwide [5], including the development of feed additives, biologically active substances (BAS), that can replace antibiotics. This has to do with the fact that despite the effectiveness of antibiotics in treating and preventing livestock health problems, they also contribute to the development of adaptation to them in pathogenic microorganisms [6].

The use of plant components as feed additives that can increase livestock productivity, stimulate reproduction, and improve the consumer properties of products is relevant and promising. Such components of the livestock diet, called “phytogenic feed additives” (phytogenics), have been widely used since the year 2000, primarily in pig and poultry farming. Interest in them is associated with the restriction on using certain synthetic growth promoters (antibiotic growth promoters) in EU countries [7]. The main reason for these limitations is the desire to improve the quality of products, and minimize the risk of developing resistance to pathogenic microorganisms both in animals and humans as the final consumers [8].

The main effects of phytogenics are increased food intake, stimulation of digestion and growth, reduced incidence of diarrhea, improved feed efficiency and productivity, and ultimately increased production profitability (Commission implementation regulation (EC) No. 131/2012).

The search for new effective phytogenic additives is a promising and relevant area of animal husbandry, especially considering modern international requirements for product safety [9].

A promising raw material for the production of biologically active substances of antimicrobial action from which to create feed additives is medicinal plants, such as *Cotinus coggygria*, *Lunaria rediviva*, *Sarothamnus scoparius*, *Eryngium maritimum*, *Goodyera repens*, *Corallorhiza trifida*, *Listera cordata*, *Dactylorhiza* *maculata*, *Platanthera chlorantha*, *P. bifolia*, etc., of the Kaliningrad region, which is characterized by a mild climate and landscape diversity. [10].

Many plants of the *Orchidaceae* genus are used in traditional medicine. Their chemical components and pharmacology have been studied for the past 15 years. *Dactylorhiza maculata* has not undergone detailed pharmacological studies. A wide range of chemical compounds has been identified in this plant, including alkaloids, bibenzyl derivatives, flavonoids, phenanthrenes, and terpenoids, which have recently been isolated from this species. These plants’ extracts and metabolites, especially those obtained from flowers and leaves, have valuable pharmacological properties. Their diuretic, antirheumatic, anti-inflammatory, anticarcinogenic, hypoglycemic, antimicrobial, anticonvulsant, relaxing, neuroprotective, and antiviral effects are particularly interesting [11,12].

It is known that *Dactylorhiza* *maculata* is mainly found in the northern and central regions of the non-chernozem zone. The biomass of the medicinal plant contains mainly rutin, quercetin, ferulic acid, hexahydroxyflavonone, and gallic acid. In addition, the composition of the medicinal plant includes essential oils and bitter substances [13,14]. This medicinal plant is used for gastritis, gastrointestinal tract diseases, colic, and a strengthening agent for gastrointestinal tract motility and bladder diseases. *Dactylorhiza* *maculata* is used as a general tonic in folk medicine, and has anti-inflammatory and enveloping properties [14].

Over the past decades, increased anthropogenic activity, rapid industrialization, and modern agricultural practices have led to increased environmental pollution with heavy metals, which has increased the toxicity of plants, including non-endemic ones. Large areas of land are already contaminated with heavy metals due to the use of pesticides, fertilizers, household and compost waste, as well as due to the emissions of heavy metals by metallurgical facilities, and mines containing metals. Although many heavy metals are found naturally in the Earth’s crust at various levels, the problem arises when they are released into the environment in excess by natural and/or anthropogenic activities and, through the air and water, enter and accumulate in plants [15].

This study aimed to investigate the properties and characteristics of the in vitro *Dactylorhiza* *maculata* extract (chemical composition, content of biologically active substances, content of heavy metals, toxicological properties, antimicrobial and antioxidant activity). For the first time, rational values of the parameters of the BAS isolation process from *Dactylorhiza maculata*, which is widespread in the territory of the Russian region of the Baltics, were determined. The content of heavy metals and toxicological properties were determined, and the antimicrobial and antioxidant activities of the *Dactylorhiza maculata* extracts were proved; the obtained data allowed the consideration of *Dactylorhiza maculata* as a possible part of feed additive formulations aiming to increase the physiological and immune status of livestock and poultry.

## 2. Results

In this study, the conditions for the production process of *Dactylorhiza* *maculata* extracts enriched with BAS were selected. The extraction temperature, duration, and hydro module (the quantitative ratio of organic solvent and plant raw materials) were chosen as the primary technological parameters that affect the yield of the plant extract of biologically active substances [14].

### 2.1. Optimization of BAS Extraction Parameters

At the preliminary stage of the study, the possibility of using different solvents (methanol, acetone, isopropyl alcohol, and ethanol) at different concentrations to obtain *Dactylorhiza* *maculata* extracts was considered. It was found that the highest extract yield is obtained when using ethanol with a concentration of 70%. The results of optimizing the technological parameters for producing a *Dactylorhiza* *maculata* extract enriched with BAS using 70% ethanol as an extractant are presented in Table 1 and Table 2.

### 2.2. Isolation of BAS from Extracts

**Figure 1:**
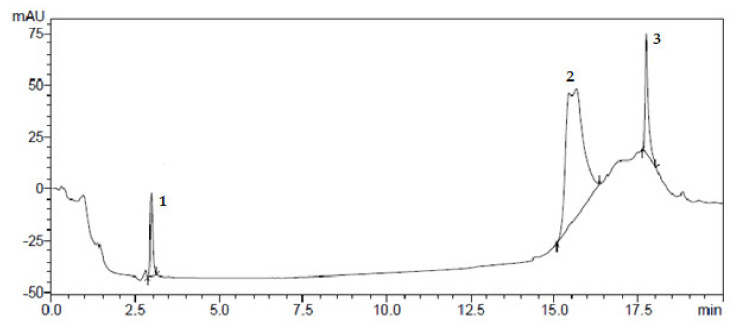
Preparative isolation of BAS from *Dactylorhiza maculata* extract samples: (1) rutin; (2) hyperoside; (3) ferulic acid.

**Figure 2:**
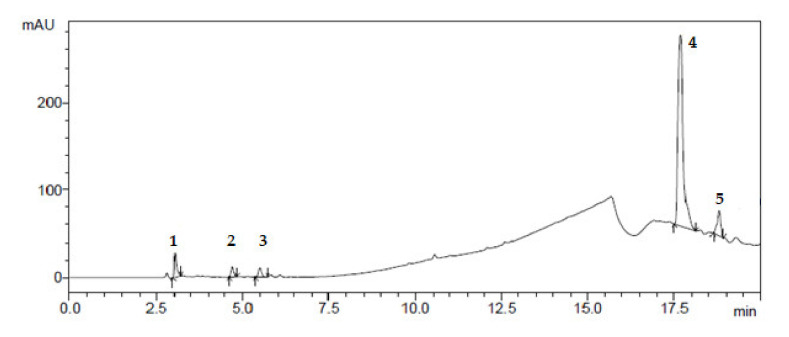
Preparative isolation of BAS from *Dactylorhiza maculata* extract samples: (1) disulforetin; (2) sulfuretin; (3) sulfurein; (4) quercetin; (5) kaempferol.

**Figure 3:**
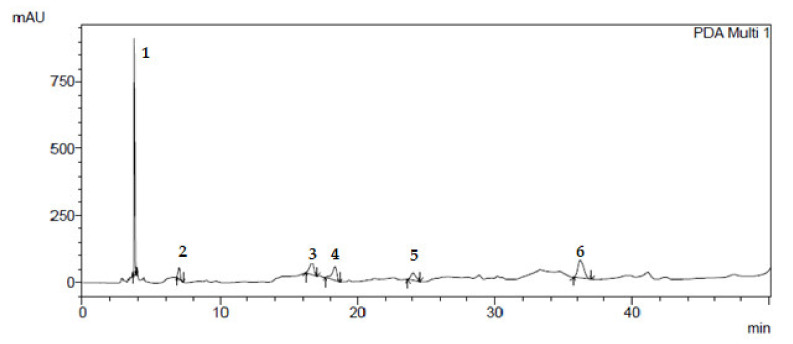
Isolation of hydroxyflavones from *Dactylorhiza maculata* extract samples by preparative chromatography: (1) apigenin; (2) 7,8-dihydroxyflavone; (3) 3-hydroxyflavone; (4) 5-hydroxyflavone; (5) luteolin; (6) luteolin 3,4′-diglucoside.

**Figure 4:**
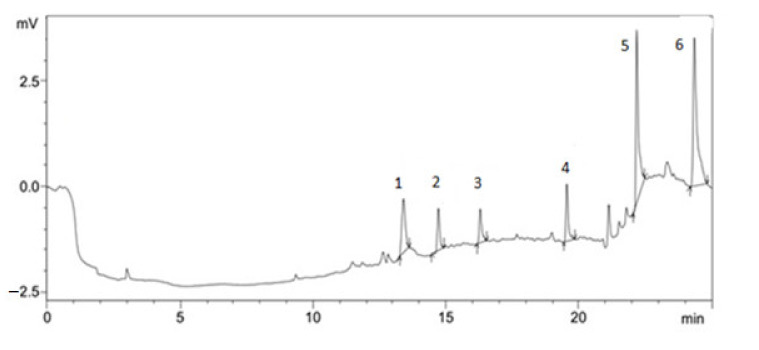
BAS isolated from *Dactylorhiza* *maculata* extract samples by HPLC: (1) 3,3′,4′,5,5′,7-hexahydroxyflavonone; (2) 3,3′,4′,5,5′,7-hexahydroxyflavonone-3-*O*-glycoside; (3) gallic acid; (4) ferulic acid; (5) rutin; (6) quercetin.

Preparative chromatography was used to isolate individual compounds from the mixture in the pure form [15]. The results of isolating individual biologically active substances from *Dactylorhiza* *maculata* extract samples by preparative chromatography are shown in Figure 1, Figure 2, Figure 3 and Figure 4.

The results of determining the qualitative and quantitative composition of BAS from *Dactylorhiza maculata* extracts are presented in Table 3.

### 2.3. Determination of the Antimicrobial Activity of Extracts

The results of evaluating the antimicrobial activity of *Dactylorhiza maculata* extracts are presented in Table 4.

The studied *Dactylorhiza maculata* extract samples had antimicrobial properties against the tested strains (*Escherichia coli, Staphylococcus aureus, Proteus vulgaris, Candida albicans, Leuconostoc. mesenteroides*).

### 2.4. Determination of Toxicological Parameters of Extracts

The content of toxic elements is presented in Table 5.

The content of heavy metals, pesticides, aflatoxin B1, and radionuclides in the *Dactylorhiza maculata* BAS complex extract samples was within the acceptable limits [12,16].

### 2.5. Determination of the Antioxidant Activity of Extracts

The antioxidant activity of the *Dactylorhiza maculata* extract samples was 217.89 ± 10.89 mg AA/g, which indicates the high antioxidant characteristics of the obtained extracts.

### 2.6. Determination of Microbiological Parameters of Extracts

The results of studying the microbiological safety indicators of the obtained *Dactylorhiza maculata* BAS complex extract samples showed their high microbiological reliability (coliform bacteria, toxin-forming anaerobes, pathogenic microorganisms, including *L. monocytogenes*, *Salmonella*, and *St. aureus* were not found).

## 3. Discussion

The extraction process was carried out at different temperatures for a duration from 10 min to 360 min (Table 1 and Table 2). No statistically significant differences (*p* < 0.05) were found in the yield of the extract, with an extraction duration of at least 60 min. With this extraction duration (≥60 min), no statistically significant differences were found at the ratio of the hydro module at 1:1, 1:2 and 1:5, 1:10, 1:20, but significant differences were established between 1:(2 maximum) and 1:(5 minimum). A statistically significant difference was found at an extraction temperature up to 40 °C and not less than 60 °C (*p* = 0.879−0.921). The recommended parameters for producing a plant extract from *Dactylorhiza* *maculata* were a temperature of 60 °C, a process duration of 60 min, and a hydro-module of 1:10. The yield of the plant extract, in this case, was 9.07%. Under these conditions, the extraction of biologically active substances was the most complete (the extraction process was carried out with an aqueous solution of ethyl alcohol with a mass fraction of the active substance of 70%) [16].

Rutin, quercetin, 3,3′,4′,5,5′,7-hexahydroxyflavonone-3-*O*-glycoside, gallic acid, and ferulic acid were isolated from fractions of *Dactylorhiza* *maculata* extract in the system with methylene chloride:methanol at 4:1 by preparative HPLC. HPLC was carried out using a column with phenyl end-capping and modified with nitrile and octyl groups, sorbent size 2.5 μm, pores 25 nm, inner diameter 2.5 mm, length 150 mm. The eluent was composed of phosphate buffer (pH 8.8) and 30% acetonitrile, with a flow rate of 1 mL/min, and a chromatography time of 30 min. The collection of individual substances was carried out automatically using a fraction collector [17].

The *Dactylorhiza* *maculata* extract (Table 3) showed high concentrations of gallic (35.92 mg/100 g) and ferulic (24.09 mg/100 g) acids. The extract of this plant also contained significant amounts of such flavonoids as 3,3′,4′,5,5′,7-hexahydroxyflavonone-3-*O*-glycoside (13.04 mg/100 g), 3,3′,4′,5,5′,7-hexahydroxyflavonone (12.58 mg/100 g), quercetin (11.07 mg/100 g) and rutin (8.52 mg/100 g).

When comparing the BAS of the *Dactylorhiza* *maculata* growing on the Kaliningrad region and the Belarus territory, it was found that in vitro *Dactylorhiza* *maculata* of the Kaliningrad region contains 13% more flavonoids than the Belarusian one [18].

As *Dactylorhiza maculata* extracts are planned to be used for producing feed additives for livestock, studying their toxicological and microbiological safety indicators was of interest [19]. It is known that the toxicological indicators of plant raw materials and extracts of the BAS complex isolated from medicinal plants are not constant and depend on the composition of the soil and the ecological situation of the growing region. Microbiological indicators of the feedstock intended for the production of feed additives for livestock are also not constant and depend on the sanitary and hygienic state of production facilities. In this regard, the toxicological and microbiological indicators of the safety of herbal extracts should be regularly studied. The main toxicological indicators were the contents of mercury, cadmium, lead, arsenic, copper, zinc, as well as the contents of aflatoxin B1, pesticides, and radionuclides.

Empirical data obtained in the course of studying the quantitative content of heavy metals, in plant extracts of the BAS complex from *Dactylorhiza maculata*, show that these extracts have a low content of such metals as mercury, cadmium, lead, and arsenic.

Thus, mercury content in the studied samples of plant extracts of the BAS complex isolated from *Dactylorhiza maculata* did not exceed 0.0001 mg/kg. The cadmium and lead content were within the normal range and was less than 0.002 mg/kg for each indicator. It was experimentally established that the arsenic content in plant extracts did not exceed 0.08 mg/kg.

The BAS complex extracts isolated from *Dactylorhiza maculata* contained copper and zinc compounds, but all indicators were within the normal range. The sum of α-, β-, and γ-isomers of hexachlorocyclohexane in the samples under study did not exceed 0.001 mg/kg, and the content of 1,1,1-trichloro-2,2-bis(4-chlorophenyl)ethane and its metabolites did not exceed 0.007 mg/kg. It was also shown that the studied plant extracts of the BAS complex were within the normal range in terms of the content of aflatoxin B1 and radionuclides. *Dactylorhiza maculata* extracts were distinguished by high microbiological reliability (coliform bacteria in 0.01 g, toxin-forming anaerobes in 1.0 g, pathogenic microorganisms, including *L. monocytogenes* and bacteria of the genus *Salmonella* in 25.0 g, *St. aureus* in 1.0 g-were not detected).

The Table 4 data indicate that the studied Dactylorhiza maculata extracts had antimicrobial properties against the tested strains (Escherichia coli, Staphylococcus aureus, Proteus vulgaris, Candida albicans, Leuconostoc mesenteroides).

The antioxidant activity of the *Dactylorhiza maculata* extract samples obtained in the territory of central Russia was 217.89 ± 10.89 mg AA/g, which indicates their high antioxidant properties.

Our results were checked against the results of studying the properties of *Dactylorhiza maculata* growing in Romania [20]. The research data show that the *D. maculata* ethanol extract (ethanol content 70%) contained the highest amount of isoquercitrin (518.6 ± 3.7 μg/g), followed by quercitrin (474.6 ± 4.34 μg/g). Ferulic acid, para-coumaric acid, caffeic acid, and kaempferol were also identified, but at a lower concentration (51.4 ± 0.45 μg/g, 69 ± 0.54 μg/g, 16.56 ± 0.12 μg/g, and 3.6 ± 0.22 μg/g, respectively. At the same time, in contrast to our extract samples, such compounds as gallic acid and such flavonoids as 3,3′,4′,5,5′,7-hexahydroxyflavonone-3-O-glycoside, 3,3′,4′,5,5′,7-hexahydroxyflavonone, and rutin were not found. The obtained data indicate that the flavonoid composition of *Dactylorhiza maculata* growing in the Russian Baltic region is much more diverse than the composition of intact *Dactylorhiza maculata* growing in Romania, containing a large set of polyphenolic compounds. The antioxidant activity presented in the studies [20] was 88.87 ± 0.9 mg AA/g, while the antioxidant activity of our *Dactylorhiza maculata* extract samples is 2.5 times higher. The plant components that prevent the accumulation of oxidants in *Dactylorhiza maculata*, which we collected, are represented by flavonoids and phenolic compounds, while in studies [20], antioxidants are represented mainly by polyphenols and vitamins C, E, and β-carotene. These facts can be explained by favorable microclimate conditions with a sufficient amount of nutrients in the soil. The obtained data determine the prospects for the use of in vitro *Dactylorhiza maculata* BAS as feed additives.

In the study of the antimicrobial activity of *Dactylorhiza maculata* extracts, presented in [20], it was found that the results of determining the lysis zone are in good agreement with our data. In our studies and in [20], antimicrobial activity was found against the same pathogenic and opportunistic microorganisms: *E. coli*, *S. aureus*, *P. vulgaris*, *C. albicans*, and *L. mesenteroides*.

The presence of antioxidant activity was proved by significant results that correlate with the contents of antioxidants (polyphenolic compounds) in *Dactylorhiza maculata*, growing in the territory of the Russian region of the Baltics. All these results represent essential criteria for the identification and characterization of biologically active substances. In addition, these studies offer new opportunities for further studies of bioactive compounds of in vitro *D. maculata* species.

The results of these studies indicate the safety of the obtained samples of the *Dactylorhiza maculata* extract from a toxicological and microbiological point of view, which makes it possible to recommend their use as ingredients in the formulation of feed additives for livestock [21].

## 4. Materials and Methods

### 4.1. Research Objects

Samples of *Dactylorhiza maculata* (L.) Soó (family *Orchidaceae*) extract were used as objects of research. The raw material for the extracts was the medicinal plant *Dactylorhiza* *maculata*, collected in August 2020 in the Kaliningrad region. The biomaterial of the species was confirmed by A. Pungin, the head of the herbarium of the Institute of Living Systems of the IKBFU (protocol No. 16/2020). The aerial parts of mature plants (shoots, leaves, and flowers) were collected. The ratio of shoots/leaves/flowers in the harvested biomass was, on average, 4:2:1 for each medicinal plant. Extracts were produced from the collected biomaterial of the medicinal plant.

### 4.2. Study of the Chemical Composition of Plant Extracts

HPLC method (Shimadzu LC-20AD chromatograph, Kyoto, Japan) according to GPM.1.2.1.2.0005.15 (high-performance liquid chromatography: general pharmacopeia monograph) was applied to study the chemical composition of *Dactylorhiza maculata* extracts; detection was carried out using a diode array detector in the range detection of 180 nm–900 nm, the flow rate of the eluent in all cases was 1 mL/min. The preparative isolation of *Dactylorhiza maculata* hydroxyflavones was carried out by HPLC, using an amine phase column with phenyl end-capping, sorbent size 2.5 μm, pores 25 nm, inner diameter 2.5 mm, length 150 mm. The conditions were: eluent phosphate buffer, pH 8.8, 30% acetonitrile, flow rate 1 mL/min, chromatography time 60 min. The collection of individual substances was carried out automatically using a fraction collector [3].

3,3′,4′,5,5′,7–hexahydroxyflavonone (≥95.0%, 42866), 3,3′,4′,5,5′,7-hexahydroxyflavonone-3-*O*-glycoside (certified reference material, CAS:153-18-4), 3-hydroxyflavone (≥98%, H4280), 5-hydroxyflavone (≥90%, H4405), 7,8-dihydroxyflavone (certified reference material, CAS:38183-03-8), apigenin (≥95%, 10798), gallic acid (≥97.5%, G7384), hyperoside (certified reference material, CAS:482-36-0), disulfuretin (certified reference material, CAS:97-77-8), quercetin (≥95.0%, Q4951), kaempferol (≥97.0%, 60010), luteolin (≥98%, L9283), ruthin (≥95.0%, PHL89270), sulfurein (certified reference material, CAS:120-05-8), sulfuretin (certified reference material, CAS: 120-05-8), and ferulic acid (≥99%, 128708) were purchased from Fluka/Sigma-Aldrich (Sigma-Aldrich Rus., Moscow, Russia). The values of the standard samples for BAS were used to construct calibration curves, which were used to determine the number of components.

### 4.3. Determination of the Heavy Metal Content in Plant Extracts

The method for the mercury content determination is based on the destruction of the analyzed sample by a mixture of nitric and sulfuric acids, precipitation of mercury with copper iodide, and subsequent colorimetric determination in the form of copper tetraiodomercuroate, by comparison with a standard scale [16].

The method for determining cadmium is based on the dry mineralization (ashing) of a sample using nitric acid as an auxiliary agent, and quantitative determination of cadmium by polarography in alternating current mode [16].

The method for arsenic determination is based on measuring the color intensity of a solution of a complex compound of arsenic with silver diethyldithiocarbamate in chloroform. The method for the determination of copper and zinc is based on the dry mineralization (ashing) of a sample using nitric acid as an auxiliary agent, and quantitative determination of copper (zinc) by polarography, in alternating current mode [2].

### 4.4. Study of Toxicological Indicators of Plant Extracts

The presence of aflatoxin B1 in the *Dactylorhiza maculata* extract was determined using the enzyme immunoassay [22]. Organochlorine pesticides were determined by gas chromatography with an electron capture detector or a mass selective detector [23]. The presence of radionuclides was determined by measuring the activity of radionuclides using the radiometric method [23].

### 4.5. Study of Microbiological Parameters of Plant Extracts

The method for the determination of mesophilic aerobic and facultative anaerobic microorganisms is based on inoculation of the product and/or dilutions of the weighed product portion into a liquid nutrient medium, the incubation of inoculates, accounting for visible signs of microorganism growth, if necessary, the subculture of culture liquid on agar nutrient media to confirm the growth of microorganisms, and finally, counting their numbers using the table. Coliform bacteria are determined by inoculation on agar selective diagnostic media; toxin-forming anaerobes are determined by inoculating a certain amount of the product or its dilutions into a liquid selective medium, isolating characteristic colonies and confirming their attribution using biochemical tests. Pathogenic microorganisms of the genus *Listeria* are determined by inoculation on a selective medium and the presence of colonial growth with growth characteristics. *S. aureus* was detected and quantified by inoculation on agar selective diagnostic media, counting the number of characteristic colonies (when determining the *S. aureus* number), confirming the attribution of the isolated characteristic colonies to *S. aureus* by biochemical signs [24].

### 4.6. Determination of the Antimicrobial Activity of Plant Extracts

Antimicrobial activity of the in vitro *Dactylorhiza maculata* extract samples against the growth of opportunistic and pathogenic test strains was determined by two methods: the diffusion method (on a solid nutrient medium) and a method based on measuring the optical density (in a liquid nutrient medium) [2]. Medical and natural test strains were used as opportunistic and pathogenic microorganisms (*Escherichia coli* ATCC 25922, *Staphylococcus aureus* ATCC 25923, *Proteus vulgaris* ATCC 63, *Candida albicans* EMTK 34, *Leuconostoc mesenteroides* EMTK 1865), acquired from the State Collection of Pathogenic Microorganisms and Cell Cultures (GKPM-Obolensk, Obolensk, Russia).

The bacterial lawns of test strains were produced on agar nutrient media, and simultaneously, BAS complex extract samples were placed on the lawn. A paper disc with a nutrient medium was used as a control, and a disc with antibiotic rifampicin (from a standard kit) was used as a reference drug. Petri dishes were incubated at a temperature corresponding to the optimal growth temperature of each test strain for 24 ± 0.5 h. The results were accounted by the presence and size (in mm) of the transparent zone showing the absence of microorganism growth around the disc [3].

All standard media were purchased from Khimreaktivsnab, Ufa, Russia.

### 4.7. Determination of the Antioxidant Activity of Plant Extracts

The antioxidant activity of plant extracts was determined by their ability to reduce the 2,2-diphenyl-1-picrylhydrazyl radical (DPPH, C_18_H_12_N_5_O_6_, M = 394.33). The reaction of the interaction of antioxidants with the DPPH radical proceeds according to the scheme [15]:

DPPH * + AH → DPPH–H + A *

As a result of the DPPH radical reduction by an antioxidant, the purple-blue color of DPPH in ethanol decreases, and the reaction is monitored by the change in optical density using conventional spectrophotometric methods.

All chemicals (analytical or higher grade) used in this study were purchased from Fluka/Sigma-Aldrich (Sigma-Aldrich Rus., Moscow, Russia).

### 4.8. Statistical Analysis

Each experiment was repeated three times, and the data are expressed as means ± standard deviation. Data processing was carried out via the standard methods of mathematical statistics. Post hoc analysis (Tukey test) was undertaken to identify samples that were significantly different from each other. The equality of the variances of the extracted samples was checked using the Levene test. The data were subjected to analysis of variance (ANOVA) using Statistica 10.0 (StatSoft Inc., 2007, Tusla, OK, USA). Differences between means were considered significant when the confidence interval was below 5% (*p* < 0.05).

## 5. Conclusions

The chemical composition of the medicinal plant *Dactylorhiza* *maculata*, growing in the Kaliningrad region, has been studied. As the result of these studies, the chemical composition, the BAS content, the content of heavy metals, toxicological properties, the antimicrobial and antioxidant activity of the in vitro plant *Dactylorhiza* *maculata* were screened for the first time. It was found that the number of flavonoids in in vitro *Dactylorhiza maculata* is not inferior to the number of flavonoids in a plant [12]. The studied *Dactylorhiza* *maculata* samples have demonstrated a broad applicability potential, with the optimal combination of antimicrobial and antioxidant activities and the content of a significant amount of essential biologically active substances.

Plant-based feed additives are now widely used to treat and prevent many diseases of livestock and poultry. A limiting factor in introducing domestic medicinal plants into the production of feed additives is the lack of information on the chemical composition of medicinal plant raw materials, and poor knowledge of the medicinal properties of plant preparations [25]. The use of medicinal plants (such as *Rhaponticum carthamoides*, *Echinacea purpurea*, *Cotinus coggygria*, *Eryngium maritimum*, *Dactylorhiza maculata*, *Platanthera chlorantha*, and many others containing similar antimicrobial and antioxidant components) for the production of feed additives is relevant to improving regional economies and improving the quality of life and the nation’s health, by providing ecologically clean livestock products. The flavonoids, which are of interest for the production of feed additives for livestock that are an alternative to antibiotics, have been identified among *Dactylorhiza* *maculata* BAS.

This study of *Dactylorhiza maculata* properties offers new opportunities for further research aimed at developing technologies for obtaining plant material in a short time, year-round, regardless of weather conditions; the possibility of producing planting material for species that are difficult to reproduce; reproduction of hybrid forms of plants with preservation of valuable properties; improvement of planting material; the possibility of automating the in vitro reproduction process, and ensuring the genetic homogeneity of the resulting plants.

## Figures and Tables

**Table 1:** Dependence of the yield of the *Dactylorhiza* *maculata* extract on the hydro module at different durations of the process (temperature 60 °C).

Hydro Module	Extract Yield (%) at the Different Duration of the Extraction Process, Min					
	10	30	60	120	180	360
1:1	1.15 ± 0.03 ^a/a^	2.14 ± 0.06 ^a/b^	6.66 ± 0.19 ^a/c^	6.92 ± 0.20 ^a/c^	7.15 ± 0.21 ^a/c^	7.90 ± 0.23 ^a/c^
1:2	1.93 ± 0.05 ^ab/a^	2.57 ± 0.07 ^ab/a^	7.19 ± 0.21 ^a/b^	7.81 ± 0.23 ^a/b^	8.12 ± 0.24 ^ab/bc^	8.71 ± 0.26 ^ab/c^
1:5	2.28 ± 0.06 ^b/a^	3.12 ± 0.09 ^b/a^	8.72 ± 0.26 ^b/b^	8.86 ± 0.26 ^b/b^	8.92 ± 0.26 ^b/b^	9.02 ± 0.27 ^b/b^
1:10	2.82 ± 0.08 ^bc/a^	4.71 ± 0.14 ^c/b^	9.07 ± 0.27 ^b/c^	9.05 ± 0.26 ^b/c^	9.03 ± 0.27 ^b/c^	9.01 ± 0.27 ^b/c^
1:20	3.07 ± 0.09 ^c/a^	4.84 ± 0.14 ^c/b^	9.15 ± 0.27 ^b/c^	9.10 ± 0.27 ^b/c^	9.03 ± 0.27 ^b/c^	9.02 ± 0.27 ^b/c^

Data presented as a mean ± SD (*n* = 3). Values in columns/rows followed by the same letter do not differ significantly (*p* > 0.05), as assessed by the post hoc test (Tukey test).

**Table 2:** Dependence of the *Dactylorhiza* *maculata* extract yield on temperature for different durations of the process (hydro module 1:10).

Temperature, °C	Extract Yield (%) at the Different Duration of the Extraction Process, Min					
	10	30	60	120	180	360
25	1.66 ± 0.05 ^a/a^	3.83 ± 0.11 ^a/b^	9.12 ± 0.27 ^a/c^	9.24 ± 0.27 ^a/c^	9.82 ± 0.29 ^a/c^	9.91 ± 0.29 ^a/c^
40	2.21 ± 0.06 ^a/a^	5.99 ± 0.18 ^b/b^	10.04 ± 0.30 ^ab/c^	10.02 ± 0.30 ^ab/c^	10.02 ± 0.30 ^ab/c^	10.05 ± 0.30 ^ab/c^
60	3.24 ± 0.09 ^b/a^	6.47 ± 0.19 ^b/b^	10.75 ± 0.32 ^b/c^	10.71 ± 0.32 ^b/c^	10.75 ± 0.32 ^b/c^	10.74 ± 0.32 ^b/c^
70	4.07 ± 0.12 ^c/a^	6.98 ± 0.21 ^b/b^	10.68 ± 0.32 ^b/c^	10.63 ± 0.32 ^b/c^	10.59 ± 0.32 ^b/c^	10.14 ± 0.32 ^b/c^
Boiling	4.19 ± 0.12 ^c/a^	6.96 ± 0.20 ^b/b^	10.57 ± 0.32 ^b/c^	10.48 ± 0.31 ^b/c^	10.33 ± 0.32 ^b/c^	10.20 ± 0.32 ^b/c^

Data presented as a mean ± SD (*n* = 3). Values in columns/rows followed by the same letter do not differ significantly (*p* > 0.05), as assessed by the post hoc test (Tukey test).

**Table 3:** The results of the quantitative determination of individual BAS from the *Dactylorhiza maculata* extract.

BAS	Content, mg/100 g
Rutin	8.52 ± 0.25
Quercetin	11.07 ± 0.33
3,3′,4′,5,5′,7-hexahydroxyflavonone	12.58 ± 0.37
3,3′,4′,5,5′,7-hexahydroxyflavonone-3-*O*-glycoside	13.04 ± 0.39
Gallic acid	35.92 ± 1.07
Ferulic acid	24.09 ± 0.72

BAS: biologically active substances. Data presented as a mean ± SD (*n* = 3).

**Table 4:** The results of evaluating the antimicrobial activity of *Dactylorhiza* *maculata* extracts.

Samples	Lysis Zone Diameter, Mm				
	*E. coli*	*S. aureus*	*P. vulgaris*	*C. albicans*	*L. mesenteroides*
Extract	17.0 ± 0.5	15.0 ± 0.5	14.0 ± 0.5	15.0 ± 0.5	17.0 ± 0.5
Control	3.0 ± 0.1	2.0 ± 0.1	2.0 ± 0.1	3.0 ± 0.1	4.0 ± 0.2
Rifampicin	23.0 ± 0.5	21.0 ± 0.5	20.0 ± 0.5	19.5 ± 0.5	18.0 ± 0.5

Control: tannin solution 1%. Data presented as a mean ± SD (*n* = 3).

**Table 5:** The content of toxic elements in *Dactylorhiza maculata* extracts.

Component	Content, mg/kg
Mercury	>0.0001
Cadmium	>0.002
Lead	>0.002
Arsenic	>0.08
Copper	0.7
Zinc	0.9
The sum of the α-, β- and γ-isomers of hexachlorocyclohexane	>0.001
1,1,1-trichloro-2,2-bis(4-chlorophenyl)ethane	>0.007
Aflatoxin B1	0.005
Strontium-90	2.0
Cesium-137	1.9

Data presented as a mean ± SD (*n* = 3).

## Data Availability

Not applicable.
